# Complication Rates After Mastectomy and Reconstruction in Breast Cancer Patients Treated with Hypofractionated Radiation Therapy Compared to Conventional Fractionation: A Single Institutional Analysis

**DOI:** 10.3390/cancers17010106

**Published:** 2025-01-01

**Authors:** Tal Falick Michaeli, Feras Hatoom, Antoni Skripai, Ella Wajnryt, Tanir M. Allweis, Shani Paluch-Shimon, Yair Shachar, Aron Popovtzer, Marc Wygoda, Philip Blumenfeld

**Affiliations:** 1 Department of Radiation Oncology, Sharett Institute of Oncology, Hadassah Medical Center, Hebrew University Medical Center, Jerusalem 91120, Israel; feras.hatoom@mail.huji.ac.il (F.H.); antoni.skripai@mail.huji.ac.il (A.S.); ellawj@gmail.com (E.W.); aron@hadassah.org.il (A.P.); mwygoda@hadassah.org.il (M.W.); philip.blumenfeld@gmail.com (P.B.); 2 Faculty of Medicine, Hebrew University, Jerusalem 91120, Israel; tanir@hadassah.org.il (T.M.A.); shanipal@hadassah.org.il (S.P.-S.); 3 Department of Military Medicine and “Tzamert”, Faculty of Medicine, Hadassah-Hebrew University of Jerusalem, Jerusalem 91120, Israel; 4 Medical Corps, Israel Defense Forces, Ramat Gan 52625, Israel; 5 Department of Breast Surgery, Hadassah-Hebrew University Medical Center, Jerusalem 91120, Israel; 6 Department of Medical Oncology, Sharett Institute of Oncology, Hadassah-Hebrew University Medical Center, Jerusalem 91120, Israel; 7 Department of Plastic Surgery, Hadassah-Hebrew University Medical Center, Jerusalem 91120, Israel; yair.shachar1@gmail.com

**Keywords:** breast cancer, PMRT, hypofractionation, breast reconstruction, implant complications

## Abstract

Introduction: Radiation therapy plays an important role in the treatment of localized breast cancer. Hypofractionated (HF) radiation therapy has emerged as a promising alternative to conventional fractionation (CF) schedules, offering comparable efficacy with reduced treatment duration and costs. However, concerns remain regarding its safety and rate of toxicity, particularly in patients undergoing mastectomy with breast reconstruction. This study aimed to assess the implant-related complications in breast cancer patients receiving HF post-mastectomy radiation therapy (PMRT) and reconstruction compared to CF PMRT. Methods: A retrospective study was conducted on 59 breast cancer patients who underwent mastectomy and breast reconstruction between 2013 and 2021 and received adjuvant PMRT. Patient demographics, treatment characteristics, and implant-related complications were analyzed. Statistical tests including chi-square, Fischer’s exact test, and multivariable Cox regression were employed for analysis. Results: Of the 59 patients, 29 received HF PMRT and 30 received CF PMRT. At a median follow-up of 23.4 months, there was no significant difference in major implant-related complications between the two groups (24.1% in HF vs. 33.3% in CF, *p* = 0.436). Most complications in the HF group occurred within the first two years post-radiation. Age over 40 was identified as a significant predictor for higher implant-related complications (*p* = 0.029). Conclusions: Our findings indicate that HF PMRT and reconstruction does not increase the risk of major implant-related complications compared to CF PMRT. These results align with the existing literature, supporting the safety of HF radiation in breast cancer patients who underwent mastectomy with reconstruction.

## Simple Summary

Our study provides valuable insights into the safety and outcomes of HF radiation therapy after mastectomy and breast reconstruction. The absence of significant differences in major implant complications between HF and CF radiotherapy groups supports the feasibility of adopting HF in this patient population. However, careful consideration of patient age and meticulous follow-up protocols remain imperative in optimizing outcomes for breast cancer patients undergoing PMRT. Of note, the limitations in the retrospective nature of our data and small number of patients may impact the generalizability of our findings. Further multi-institutional studies with extended follow-up periods could enhance our understanding of the long-term implications and benefits of HF radiotherapy in this specific clinical setting.

## 1. Introduction

Breast cancer is the most common malignancy affecting women worldwide, demanding ongoing advancements in its management [1,2]. Among the treatment modalities, adjuvant postoperative radiation therapy stands as a critical component, aiding in reducing the risk of local recurrence and improving overall survival rates for breast cancer patients [1,2,3,4,5,6]. Traditionally, radiation was delivered using conventional fractionation (CF) schedules, administering daily doses of 1.8 to 2 Gy per fraction over five to six weeks. However, a paradigm shift has occurred, favoring the adoption of hypofractionated (HF) radiation therapy for most patients undergoing whole-breast radiotherapy. This approach involves higher-radiation doses per session over a shorter three-to-four-week treatment duration, offering equivalent oncologic outcomes while reducing patient burden and healthcare costs [1,2,7,8].

For patients undergoing mastectomy, breast reconstruction serves as an essential aspect of post-mastectomy care, providing both physical and psychological benefits to patients [5,9,10], and immediate reconstruction is the preferred approach. Different options, such as implant-based reconstruction and autologous-based reconstruction, are available, allowing for flexibility depending on the timing of reconstruction (immediate or delayed), patient habitus, and preferences. However, breast reconstruction procedures carry the potential for complications including infection, flap ischemia, necrosis, and capsular contracture [9]. Post-mastectomy radiotherapy (PMRT) is often indicated to improve oncologic outcomes [1,2,3,4,5,6]. However, it can also increase complications and contribute to poorer esthetic results in patients who underwent breast reconstruction [5,9].

In recent years, several studies have been published exploring the safety of HF PMRT, both with and without reconstruction [1,3,9,11,12]. However, concerns about treating patients with HF PMRT after breast reconstruction still persist. At our institution, breast cancer patients who underwent mastectomy and reconstruction have been treated with both fractionation schemas. This study aims to investigate the oncologic outcomes and risk of complications in breast cancer patients who underwent mastectomy and breast reconstruction and were treated with HF versus CF PMRT.

## 2. Methods

### 2.1. Study Design and Patients

We conducted a retrospective, single-institution study including 59 consecutive breast cancer patients who underwent mastectomy with immediate or delayed breast reconstruction and who received adjuvant RT between the years 2013 and 2021 at Hadassah Medical Center. The study adhered to the Declaration of Helsinki and was approved by the institution’s ethics committee.

### 2.2. Inclusion Criteria

The study included breast cancer patients who underwent mastectomy and breast reconstruction and received adjuvant RT between the years 2013 and 2021.

### 2.3. Exclusion Criteria

Patients who had implant-related complications before receiving RT were excluded from the study.

### 2.4. Demographics and Characteristics

Patient demographics and characteristics were assessed to investigate their potential impact on the risk of developing implant-related complications. Variables included body mass index (BMI), history of smoking, history of diabetes mellitus, tumor staging, hormonal status of the tumor, whether adjuvant or neoadjuvant chemotherapy was administered, and the type of reconstruction surgery performed.

### 2.5. Definitions of Implant-Related Complications

The primary toxicity endpoint was implant-related complications leading to re-hospitalization or implant failure, including capsular contracture, skin necrosis, implant rupture, and infection. Complications that did not cause re-hospitalization, reoperation, or implant failure were not included in the analysis.

### 2.6. Breast Reconstruction

All patients underwent either immediate or delayed breast reconstruction surgery, with three types of breast reconstruction procedures performed: autologous (such as latissimus dorsi myocutaneous flap and deep inferior epigastric perforator (DIEP) free flap) or alloplastic (implant-based reconstruction), either direct to implant or two-staged expander-implant reconstruction.

### 2.7. Radiotherapy

For both HF and CF PMRT, a 3D conformal tangential field technique was employed with field-in-field when necessary. An additional supraclavicular field was added when clinically indicated. Computed tomography (CT) scans were acquired for treatment planning, and the target volume and critical structures, such as the heart and lungs, were delineated by a radiation oncologist. The post-mastectomy chest wall and regional lymph nodes (when indicated) were included in the target volume. 

For HF RT, a total dose of 42.4 Gy was delivered in 16 fractions of 2.65 Gy, five days per week, over a three-week period. As for CF RT, a total dose of 50 Gy was administered in 25 fractions of 2 Gy, five days per week, over a five-week period. In cases of positive or very close margins, a 10 Gy in 4 fraction boost was delivered. Both treatment techniques were delivered using linear accelerators, and daily image guidance was performed to ensure accurate setup and target coverage. In our institution, the decision to deliver HF as compared to CF was at the discretion of the treating radiation oncologist. As expected, more patients received HF regimen from 2017 and onwards compared to prior to 2017.

During treatment, patients were closely monitored for acute toxicities, and any deviations from the treatment plan were documented and managed accordingly. Follow-up assessments were conducted regularly with medical and surgical oncology. Long-term toxicities, including implant-related complications, were evaluated and documented during follow-up visits. 

### 2.8. Statistical Analysis

Patient characteristics between the two fractionated radiotherapy (RT) regimens (HF RT vs. CF RT) were compared using the Chi-square test and Fisher’s exact test. The Kaplan–Meier method was applied to compare complication incidence rates, accounting for the significant difference in follow-up periods between the two regimens. Factors influencing any breast complications and major breast complications were analyzed using a multivariable Cox regression model. All statistical analyses were performed using IBM SPSS Statistics (version 28). A *p*-value of <0.05 was considered statistically significant.

## 3. Results

Of the 59 patients, 29 patients were treated with HF and 30 patients with CF RT. Of the 59 patients, all except 1 received radiation to both the chest wall and the draining lymphatics. Immediate reconstruction patients received PMRT after surgery, while delayed reconstruction patients received RT prior to the second surgery (range: 6–36 months).

The median age of patients was 42 years old (range: 28–72), 27 (45.8%) of the patients were below 40 years old, and 32 (54.2%) were above. Further, 27 (45.8%) of the patients had a BMI that was equal or less than 25, and 32 of the patients (54.2%) had a BMI > 25. Of the 59 patients, 4 were smokers (6.8%) and 5 had diabetes mellitus (8.5%). The median follow-up was 23.4 months (range 1.2–110.9), the median follow-up of the HF group was 18.26 months, while the median follow-up of the CF group was 42.9 months. Follow-up was defined as the time of completing radiation to complications or the last known encounter with a physician that examined the reconstructed breast. 

Among the 59 patients, 29 received HF PMRT (49.2%) and 30 received CF PMRT (50.8), 57 patients had radiation to the regional lymph nodes (96.6), and only 3 patients received a boost. Of the 59 patients, 45 patients underwent immediate reconstruction (76.3%) and 14 underwent delayed reconstruction (23.7%). There was no significant difference in terms of the operation type between the HF PMRT group and the CF PMRT group, other than the fact that the 2-stage alloplastic implant was used more commonly in the CF PMRT group [8] in comparison to the HF PMRT group [2]. Further, 30 patients (50.8%) received neoadjuvant chemotherapy, 23 (39%) received adjuvant chemotherapy, and 6 (10.2%) received hormonal therapy alone. Additionally, 53 of the 59 patients received endocrine therapy. There was no significant difference in any of the demographics listed above between the two groups, other than what is mentioned. The full demographics of the patients are shown in Table 1.

### Comparing the Two Fractionation Groups

Of the patients treated with HF PMRT, 7 patients (24.1%) had a major implant-related complication compared to 10 patients (33.3%) of the CF PMRT group. Statistical analysis showed no significant difference in the occurrence of major implant-related complications between the two groups, (*p* = 0.436). The most common complication was capsular contracture in both groups, (5 and 7 patients, respectively). Table 2 includes major complication types and rates.

**Figure 1:**
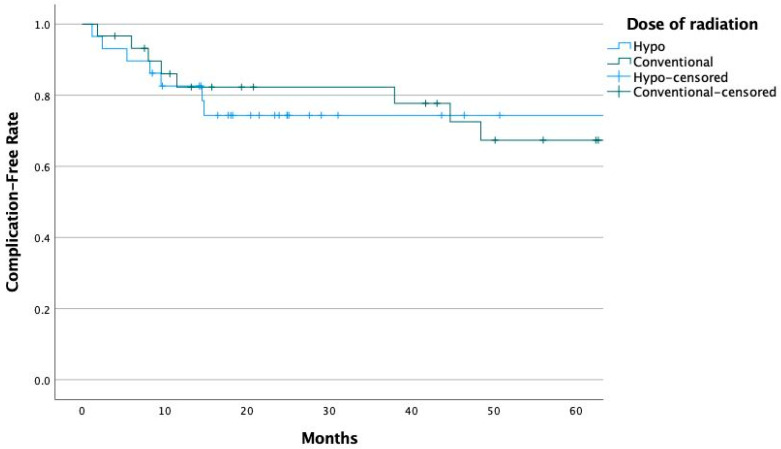
Freedom from breast complications.

Figure 1 shows Kaplan–Meir curves of freedom from breast complications comparing the two groups. Using a log rank test of the two groups in order to take into consideration the difference in the follow-up time, there was no significant difference between the two groups (*p* = 0.721).

**Figure 2:**
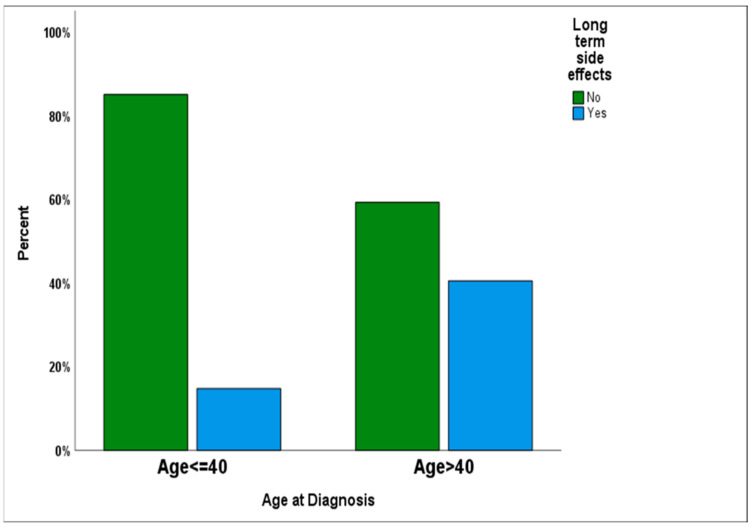
Comparing age groups.

We carried out a multivariate analysis. Out of the various variables that we analyzed, including the type of the reconstruction surgery, none had any significant impact on the complication rate other than age. We found that age > 40 was a major predictive factor for higher implant-related complications in the two groups (*p* = 0.029). Table 3 includes the variables we analyzed in the study, and Figure 2 demonstrates the percent difference in side effects between the two age groups.

**Figure 3:**
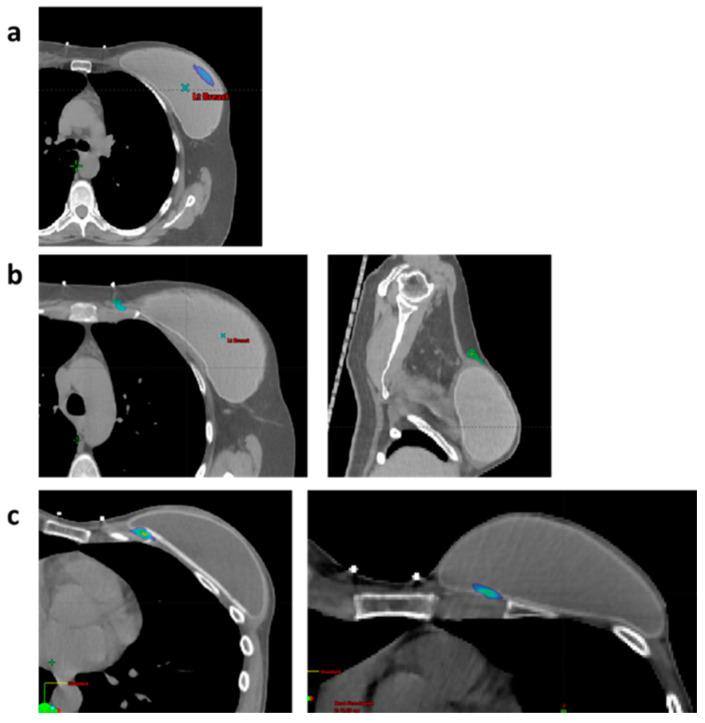
Hotspot type with respect to the reconstructed breast. Representative CT scans demonstrating three types of hotspots: inside the implant (**a**), outside the implant (**b**), and overlap respective to the implant and chest wall (**c**).

Next, we focused on the radiation properties that might have contributed to implant-related toxicity, including irradiated volume and max dose to the implant and its location in the radiation field (Table 4, Figure 3). One can see that most of the hotspot types in which toxicity developed were located somewhere in the implant. However, when comparing to the patients who did not develop any side effects, we witness a similar pattern of hotspot distribution within the implant (*p* = 0.73, NS). In that case, from our limited experience, we could not draw any conclusion based on radiation properties under the guidelines of treatment.

## 4. Discussion

Adjuvant radiation therapy, a major component in reducing the risk of local recurrence and improving overall survival rates for breast cancer patients, has witnessed a paradigm shift towards HF thanks to the widespread adoption of the START A and B and Canadian regimens in breast conservation treatment. This approach offers equivalent oncologic outcomes while reducing patient burden and healthcare costs [1,2,7,8]. As for the PMRT setting, a prospective randomized study found that adjuvant radiation with HF was found to be an equivalent option for women undergoing mastectomy without reconstruction compared to CF PMRT [1]. Given the significant rates of capsular contracture and implant loss after PMRT with CF [13], there is a concern that HF could further increase the risk of implant-related toxicity. Our study indicates that patients who received HF PMRT and reconstruction do not have an increased risk of complication compared to those undergoing CF PMRT.

There are limited prospective data to guide us as to the correct fractionation scheme in patients treated with PMRT after reconstruction. Recently, the Mayo clinic published their findings of 82 breast cancer patients who received proton PMRT to either CF (50 Gy in 25 fractions) or HF (40 Gy in 15 fractions). At 2 years, the rate of complications was 15% with CF and 20% with HF out of all the patients. Respectively, 27 and 30 patients had reconstruction. The study was not able to demonstrate non-inferiority of HF proton PMRT. The study demonstrated all complications occurred in patients who had immediate reconstruction [12]. In our cohort, which was treated with photon-based PMRT, we observed similar complication rates in reconstructed patients: 23% vs. 20% for HF and 30% vs. 33% for CF. While photon and proton therapies appear to yield comparable rates of complications in this context, proton therapy offers potential advantages in reducing radiation exposure to surrounding healthy tissues such as the heart or lungs due to its unique physical properties, particularly the Bragg peak. Future investigations should explore whether the dosimetric benefits of proton therapy translate into clinically meaningful reductions in long-term toxicity or improved outcomes for patients undergoing PMRT with reconstruction.

The type of reconstruction significantly impacts implant failure and complication rates. Implant-based reconstruction, involving the insertion of a foreign body into the breast, is associated with higher infection rates and an implant failure or contracture rate of 15–40%. Recently, there has been growing interest in prepectoral (PP) implant placement as an alternative to traditional submuscular (SM) placement to minimize complications associated with pectoralis major muscle (PMM) manipulation. A retrospective study comparing PP and SM implant-based reconstruction in breast cancer patients treated with neoadjuvant chemotherapy demonstrated no significant differences in overall complication rates or implant loss between the two approaches, while PP placement reduced operative time and avoided PMM-related morbidities [14]. Additionally, the timing of reconstruction affects cosmetic outcomes; for example, autologous reconstruction after PMRT may lead to flap asymmetry or shrinkage, although these issues are generally less pronounced [15] and they are less reported complications. In our study, we did not observe significant differences in complication rates between reconstruction types, likely due to our limited sample size. Poppe and colleagues reported favorable acute and late toxicity outcomes in patients receiving PMRT with HF at a dose of 42.56 Gy in 16 fractions. In their cohort of 43 patients with reconstruction (80% tissue expanders, 7% immediate implants), grade 3–4 complications were observed in 35%, a rate comparable to conventional fractionation (CF) and similar to our cohort [7].

Our study performed a multivariate analysis to determine predictors of high-grade complications, demonstrating that patients above the age of 40 exhibited an increased risk even when controlling for smoking and BMI, highlighting the importance of personalized risk assessment and post-treatment surveillance, particularly in older cohorts. This is in agreement with a retrospective Korean study [9] that also aimed to investigate whether HF adjuvant PMRT increased breast-related complications compared to CF PMRT in reconstructed breast cancer patients. Their study included 349 patients from two institutions who underwent immediate breast reconstruction following mastectomy or breast-conserving surgery (BCS) and similarly to our study they have demonstrated that HF PMRT did not increase the risk of major breast complications in reconstructed breast patients; furthermore, their multivariate analysis found age > 45 to be a negative predictive factor for higher incidence of major breast complications in patients with mastectomy. In addition, they also found that implant-based reconstruction is a negative predictive factor. We were unable to detect such differences, likely due to our small sample size.

Recently, the CHARM trial randomized 898 women receiving PMRT after breast reconstruction to CF or HF. Reconstruction timing varied, with 55% undergoing delayed reconstruction and 45% having immediate reconstruction. Additionally, 57% had implant-only reconstruction while 43% had autologous-based reconstruction [15]. At two years, the rate of reconstructive complications was 12% with CF and 14% with HF, meeting the predefined non-inferiority threshold. Notably, autologous reconstruction had half the complication rate of implant-only reconstruction, and single-stage reconstruction showed benefits regardless of whether it was performed upfront or delayed. The FABREC study also demonstrated that the physical well-being and toxicity profile of HF PMRT were comparable to CF PMRT [16]. HF was associated with improved quality of life (QOL) in some domains at six months, particularly among younger patients. Consistent with these findings, our data also suggest that younger patients experience fewer side effects. Overall, these trials strongly support our single-institution retrospective experience. The growing evidence in the field for HP PMRT cosmesis non-inferiority to CV led to ESTRO recommendation for HP PMRT regardless of the timing type of reconstruction. This recommendation holds a consensus of 86.9%.

## 5. Conclusions

Our study provides valuable insights into the safety and outcomes of HF radiation therapy after mastectomy and breast reconstruction. The absence of significant differences in major implant complications between HF and CF radiotherapy groups supports the feasibility of adopting HF in this patient population. However, careful consideration of patient age and meticulous follow-up protocols remain imperative in optimizing outcomes for breast cancer patients undergoing PMRT. Of note, the limitations of the retrospective nature of our data and small number of patients may impact the generalizability of our findings. Further multi-institutional studies with extended follow-up periods could enhance our understanding of the long-term implications and benefits of HF radiotherapy in this specific clinical setting.

## Figures and Tables

**Table 1:** (a): Patient and tumor characteristics. (b): treatment characteristics.

(a)			
Characteristics	HF PMRT (n = 29)	CF PMRT (n = 30)	*p*-Value
Age			0.796
<40	14 (48.3%)	13 (43.3%)	
>40	15(51.7%)	17 ( 56.7%)	
Body Mass Index			0.195
<25	16 (55.2%)	11(36.7%)	
>25	13 (44.8%)	19 (63.3%)	
Smoker			0.353
Yes	3 (10.3%)	1 (3.3%)	
No	26 (89.6%)	29(96.6%)	
Diabetes Mellitus			1
Yes	2 (6.9%)	3 (10.0%)	
No	27 ( 93.1%)	27 ( 90.0%)	
Histology			NA
IDC	26 (89.7%)	27 (90%)	
Other	3 (10.3%)	3 (10%)	
Molecular type			NA
Luminal A	13(44.8%)	22( 73.3%)	
Luminal B	11( 37.9%)	7 (23.3%)	
HER2 enriched	4 (13.8%)	1 (3.3%)	
TNBC	1 (3.4%)	0	
**(b)**			
**Characteristics**	**HF PMRT (n = 29)**	**CF PMRT (n = 30)**	***p*-Value**
Adjuvant chemotherapy			NA
Yes	9 (31.0%)	13 (43.3%)	
No	20 (69.0%)	17(56.7)	
Neoadjuvant chemotherapy			NA
Yes	17(58.6%)	13(43.3%)	
No	12(41.4%)	17(56.7%)	
Timing of reconstruction			0.36
Immediate	24(82.75%)	21(70.0%)	
Delayed	5(17.25%)	9(30%)	
Radiation Boost			0.612
Yes	2(6.9%)	1(3.3%)	
No	27(93.1%)	29(96.7%)	
Type of surgery			0.167
Direct to alloplastic implant	21(72.4%)	15(50.0%)	
Two-stage alloplastic implant	2(6.9%)	6(20.0%)	
Autologous	6(20.7%)	9(30.0%)	

**Table 2:** Major complication types and rates.

Complication Type	CF PMRT (n = 30)	HF PMRT (n = 29)	*p* Value
Capsular contracture	7 (23.3%)	5 (17.2%)	
Skin necrosis	1 (3.3%)	1 (3.4%)	
Infection	1 (3.3%)	0	
Infection + capsular contracture	1 (3.3%)	1 (3.4%)	
Total	10 (33.3%)	7 (24.1%)	0.436

**Table 3:** Prognostic factors for any breast complication.

Variable	Confidence Interval	Hazard Ratio	*p*-Value
CF vs. HF PMRT	0.251–2.259	0.754	0.436
Age (over 40 vs. 40 and under)	1.237–12.093	3.868	0.029
Smoker vs. Nonsmoker	0.092–6.025	0.743	1
Delayed vs. Immediate Reconstruction	0.076–2.105	0.399	0.31
Radiation Boost vs. no boost	NA	NA	0.55
BMI (≤25 vs. >25)	0.076–2.105	1.184	0.899

**Table 4:** Univariable analysis on implant-related long-term complications.

Radiotherapy Properties	Complications Developed	
	Yes	No
Average PTV volume (cc)	836.2 (422.5–1455)	760.6 (357–1793)
Average Max dose (%)	108.8 (107.2–109.9)	109.5 (107.5–112.5)
Hotspot type on implant (Number)		
Outside	3	5
Inside	4	5
Overlap	4	12
Inside + Overlap	3	4

## Data Availability

The paper shares all of the data used for analyzing the results.

## Abbreviations

CF	Conventional Fractionation
HF	Hypofractionated
PMRT	Post-Mastectomy Radiation Therapy
PTV	Planning Target Volume
